# Hadrosauroid eggs and embryos from the Upper Cretaceous (Maastrichtian) of Jiangxi Province, China

**DOI:** 10.1186/s12862-022-02012-x

**Published:** 2022-05-09

**Authors:** Lida Xing, Kecheng Niu, Tzu-Ruei Yang, Donghao Wang, Tetsuto Miyashita, Jordan C. Mallon

**Affiliations:** 1 State Key Laboratory of Biogeology and Environmental Geology, China University of Geosciences, Beijing, 100083 China; 2 School of the Earth Sciences and Resources, China University of Geosciences, Beijing, 100083 China; 3 Yingliang Stone Natural History Museum, Nan’an, 362300 China; 4 Institute of Vertebrate Paleontology and Paleoanthropology, Chinese Academy of Sciences, Beijing, 100044 China; 5 National Museum of Natural Science, Taichung, Taiwan; 6 Beaty Centre for Species Discovery and Palaeobiology Section, Canadian Museum of Nature, Ottawa, ON K1P 6P4 Canada; 7 Department of Earth Sciences, Carleton University, Ottawa, ON K1S 5B6 Canada

**Keywords:** Hadrosauroidea, Fossil eggs, Fossil embryos, Upper Cretaceous, Hekou Formation, China, Ganzhou Basin

## Abstract

**Background:**

Dinosaur eggs containing embryos are rare, limiting our understanding of dinosaur development. Recently, a clutch of subspherical dinosaur eggs was discovered while blasting for a construction project in the Upper Cretaceous red beds (Hekou Formation) of the Ganzhou Basin, Jiangxi Province, China. At least two of the eggs contain identifiable hadrosauroid embryos, described here for the first time.

**Results:**

The eggs, attributable to Spheroolithidae indet., are thin-walled and small (~ 660 mL) compared to those of Lambeosaurinae. The shape of the embryonic squamosal is reminiscent of that seen in the Late Cretaceous hadrosauroids *Levnesovia transoxiana*, *Tanius sinensis*, and *Nanningosaurus dashiensis*, suggestive of possible affinities.

**Conclusion:**

The small size of the eggs and embryos, similar to those of Hadrosaurinae, indicates that the larger eggs and hatchlings typical of Lambeosaurinae are evolutionarily derived.

## Background

Dinosaur eggs are common worldwide, but embryos are rare [1]. Among the diverse duck-billed dinosaurs and their nearest relatives (Hadrosauroidea), just three identifiable species are known from perinatal material: *Hypacrosaurus stebingeri* [2], *Maiasaura peeblesorum* [3, 4], and *Saurolophus angustirostris* [5]. Accordingly, the early ontogeny of hadrosauroids is poorly understood, which hinders determination of skeletal development and allometric trends across the clade.

Recently, a construction project in the Upper Cretaceous red beds of the Ganzhou Basin, Jiangxi Province, China revealed a fossilized clutch of spheroolithid eggs. In this contribution, we briefly describe two of these eggs and their embryonic contents, accessioned at the Yingliang Stone Natural History Museum (YLSNHM) in Fujian Province, China. The embryos (YLSNHM 01328 and 01373) share several features in common with hadrosauroids, but otherwise lack the more derived features present in embryonic hadrosaurids. The squamosal is distinctive and recalls that of some other Late Cretaceous hadrosauroids, the implications of which we explore here. We end this contribution with a consideration of the evolutionary and taphonomic implications of these eggs and their contents.

### A comment on taxonomy

Hadrosauridae is traditionally split into two subfamilies, the solid-crested Hadrosaurinae and hollow-crested Lambeosaurinae [6], the former named after the eponymous *Hadrosaurus foulkii*. However, in a relatively recent phylogenetic analysis, Prieto-Marquez [7] recovered *Hadrosaurus* as the sister taxon to all other hadrosaurids, and so, abiding by the regulations of the International Code of Zoological Nomenclature, renamed the clade of solid-crested hadrosaurids Saurolophinae. Although this new name has gained some acceptance in the literature (e.g., [8–10]), not all current phylogenies recover *Hadrosaurus* apart from the solid-crested hadrosaurids (e.g., [11, 12]). Therefore, in keeping with tradition and some recent phylogenies, we use the clade name Hadrosaurinae, which is practically synonymous with Saurolophinae, except for the inclusion of *Hadrosaurus* [13].

### Geological provenance

**Fig. 1:**
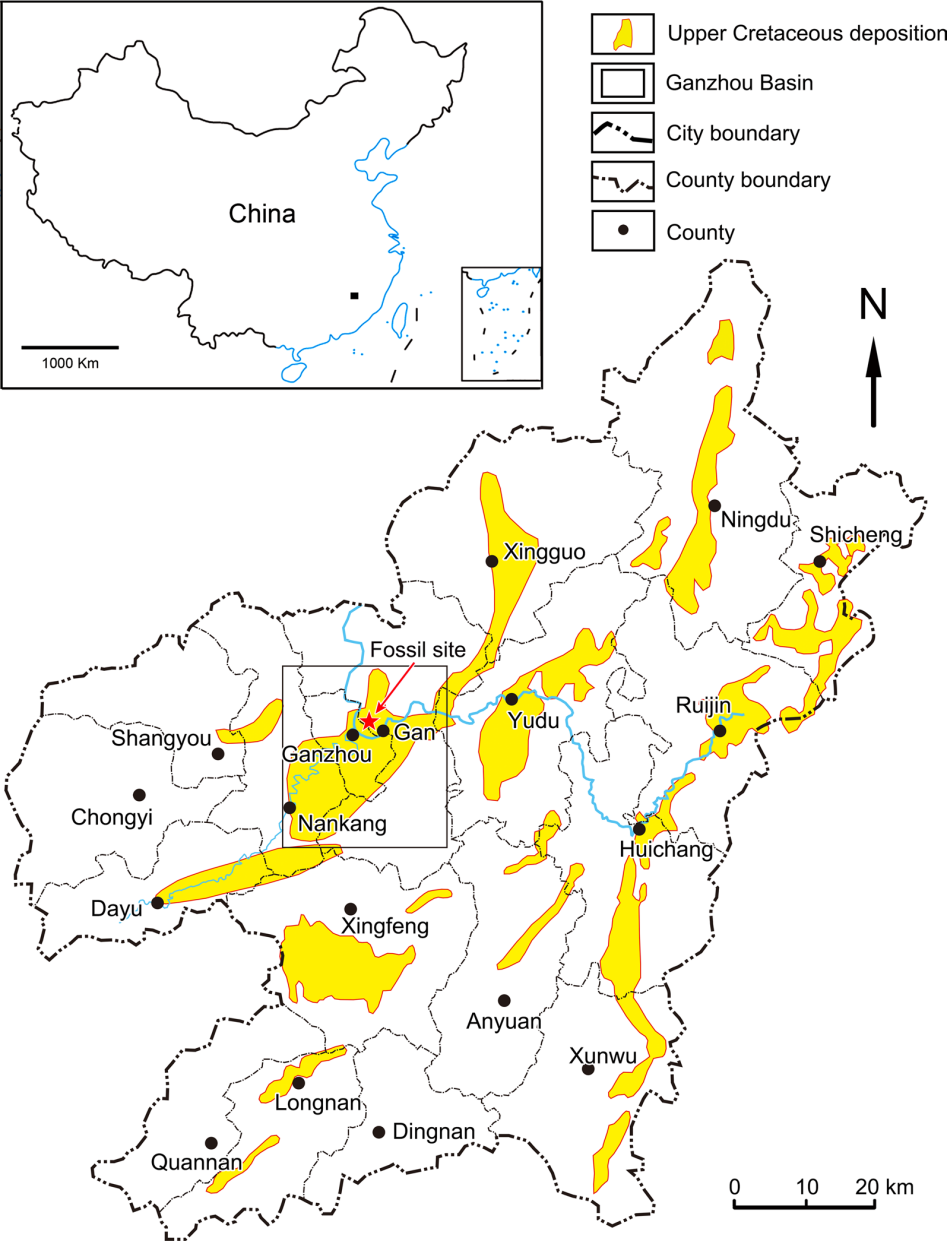
Map showing location of the embryo-bearing egg specimens, YLSNHM 01328 and 01373 (“Fossil site”) in southern China

The red beds of the Ganzhou area of Jiangxi Province are divided into: (1) the Upper Cretaceous Ganzhou Group, consisting of the Maodian and Zhoutian formations, and (2) the Cretaceous-Paleogene Guifeng Group, consisting of the Upper Cretaceous Hekou and Tangbian formations and the Cretaceous-Paleogene Lianhe Formation [14]. The eggs and embryos reported here come from the Hekou Formation of the Guifeng Group in the Ganzhou area (Fig. 1).

The Hekou Formation varies in thickness between 200 and 1600 m, depending on where it is exposed. It consists of channelized, fining-upward conglomerates, sieve deposits, and alternating sandstone and mudstone beds. The depositional setting is interpreted as primarily fluvially-derived within a proximal alluvial fan system. Mudcracks and caliche deposits are common, and the prevailing palaeoclimate is interpreted as subhumid [15].

The age of the Hekou Formation is debated. Some have argued for a Coniacian-Santonian age for the formation [16], but a Maastrichtian age is more commonly accepted. The latter interpretation is based partly on palaeomagnetic studies that have dated the host Guifeng Group to 71.4–65.0 Ma [17, 18]. Some have also argued that the Guifeng Group is penecontemporaneous with the red beds of the nearby Nanxiong Group [19], which is purportedly of Maastrichtian age [20]. In this model, the Hekou Formation is thought to correlate with the Dafeng and Yuanpu formations of the lower Nanxiong Group [21]. Indeed, the preserved theropod assemblage of the Nanxiong Group, including alioramine tyrannosaurids and oviraptorosaurs [22, 23], agrees well with the dinosaur assemblage of the Nemegt Formation in Mongolia, which itself is often considered Maastrichtian in age [24]. For these reasons, and in keeping with the recent literature (e.g., [25, 26]), we accept a probable Maastrichtian age for the Hekou Formation.

The Hekou Formation has thus far yielded fossil algae, plants, dinosaur bones, and trackways, few of which have been formally described [14, 25–29]. Dinosaur eggs assigned to *Oolithes* sp., *Oolithes spheroides* [30], “*Spheroolithus minor*”, *Ovaloolithus* sp., *Paraspheroolithus* sp., *Macroolithus rugustus*, Coelurosauria fam. et gen. indet. [27], and Elongatoolithidae [26] are also known from the Hekou Formation.

## Results

### Description of eggs

Each embryo described here is preserved within its respective egg, both of which came from the same egg clutch. Field records note the presence of at least 13 eggs in the clutch at the time of excavation, but the original number may have been higher (S Miao, pers. comm. to LX, 2008).

**Fig. 2:**
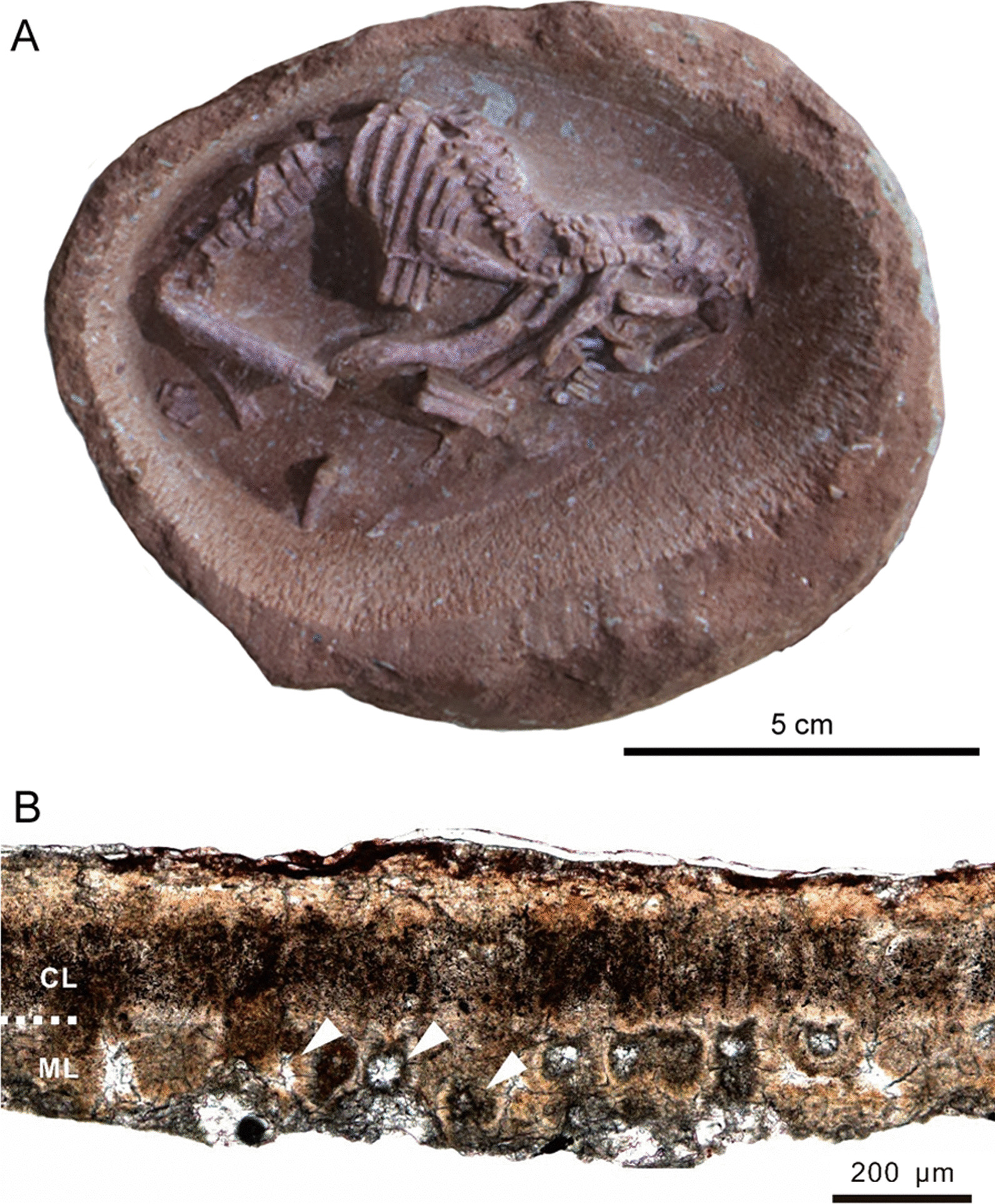
Eggshell of Spheroolithidae sp. (YLSNHM 01373). **A** Overview of egg containing embryonic hadrosauroid; **B** cross-section of the YLSNHM 01373 eggshell under transmitted, unpolarized light. The dotted line marks the boundary between the mammillary (ML) and continuous (CL) layers. The white arrows indicate the locations of organic cores

The better-preserved egg of YLSNHM 01373 has the shape of a prolate spheroid (Fig. 2A), comparable to the spherical-subspherical eggs assigned to the oofamily Spheroolithidae [31], which has been reported previously from the Hekou Formation as “*Spheroolithus minor*” [27], a nomen nudum. Given the cross-sectional dimensions of the egg, we estimate a total egg volume of approximately 600 mL. Viewed in cross-section, the embryo fills approximately 40% of the egg by area.

The eggshell of YLSNHM 01373 is poorly preserved and thus hinders our detailed description and further assignment to an oogenus. While hadrosaurids have long been associated with spheroolithid eggs based on their embryonic contents [2, 4, 32, 33], the association remains questionable due to the poor preservation of the eggshell and the absence of detailed description of the eggshells in previous reports [e.g., 2, 4]. The YLSNHM 01373 egg reveals an indistinct boundary between the mammillary and continuous layers (Fig. 2B), indicating its two-layered structure—a common microscopic feature for dinosaurian eggs. The organic cores (nucleation site for the acicular radial crystal growth in the lower part) are poorly circumscribed, probably due to severe embryo-induced erosion or poor preservation (or both). The radial crystals merge into a tabular structure in the external quarter of the eggshell. The eggshell of YLSNHM 01373 ranges 0.32–0.42 mm thick, among the thinnest known for the oofamily [34].

### Description of embryos

#### YLSNHM 01328

**Fig. 3:**
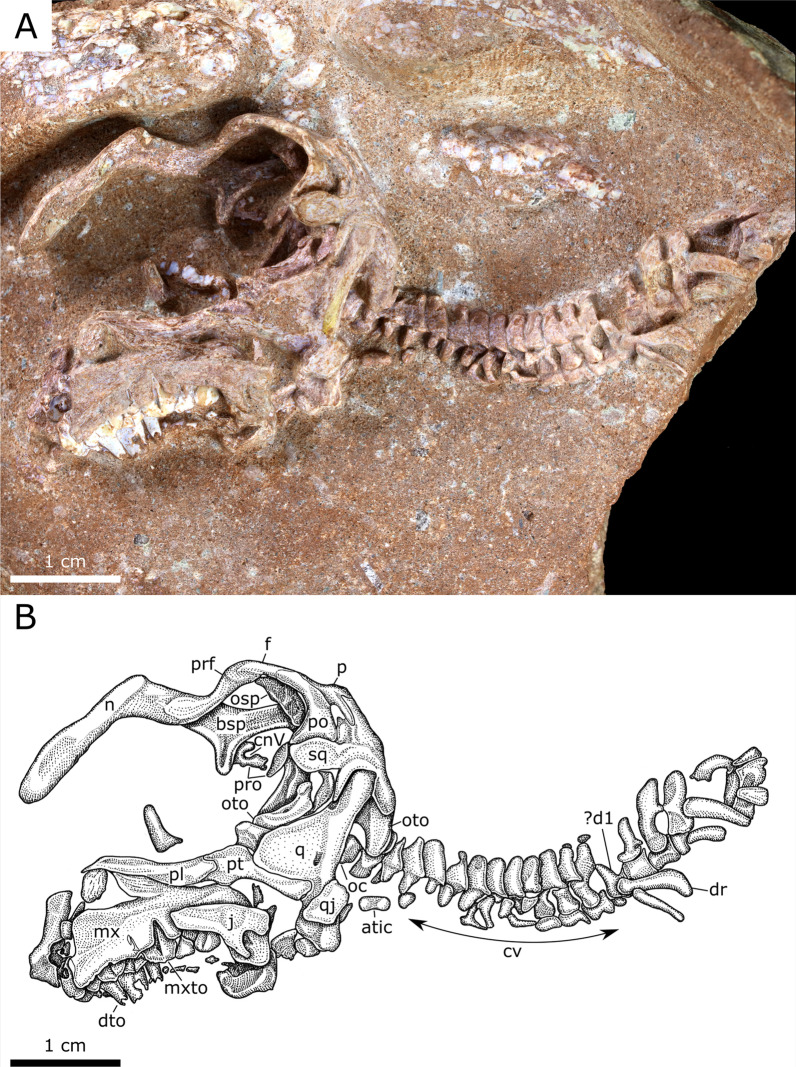
Hadrosauroid partial embryonic skeleton (YLSNHM 01328). **A** Photograph, **B** interpretive drawing. See text for list of abbreviations

**Fig. 4:**
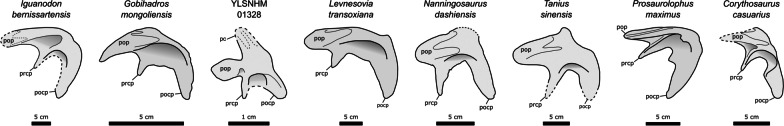
Hadrosauriform squamosals in left lateral view. *Iguanodon bernissartensis* after [58], *Gobihadros mongoliensis* after [38], *Levnesovia transoxiana* after [36], *Nanningosaurus dashiensis* after [37], *Tanius sinensis* after [35], *Prosaurolophus maximus* after [39], *Corythosaurus casuarius* after [40]. See text for list of abbreviations

**Fig. 5:**
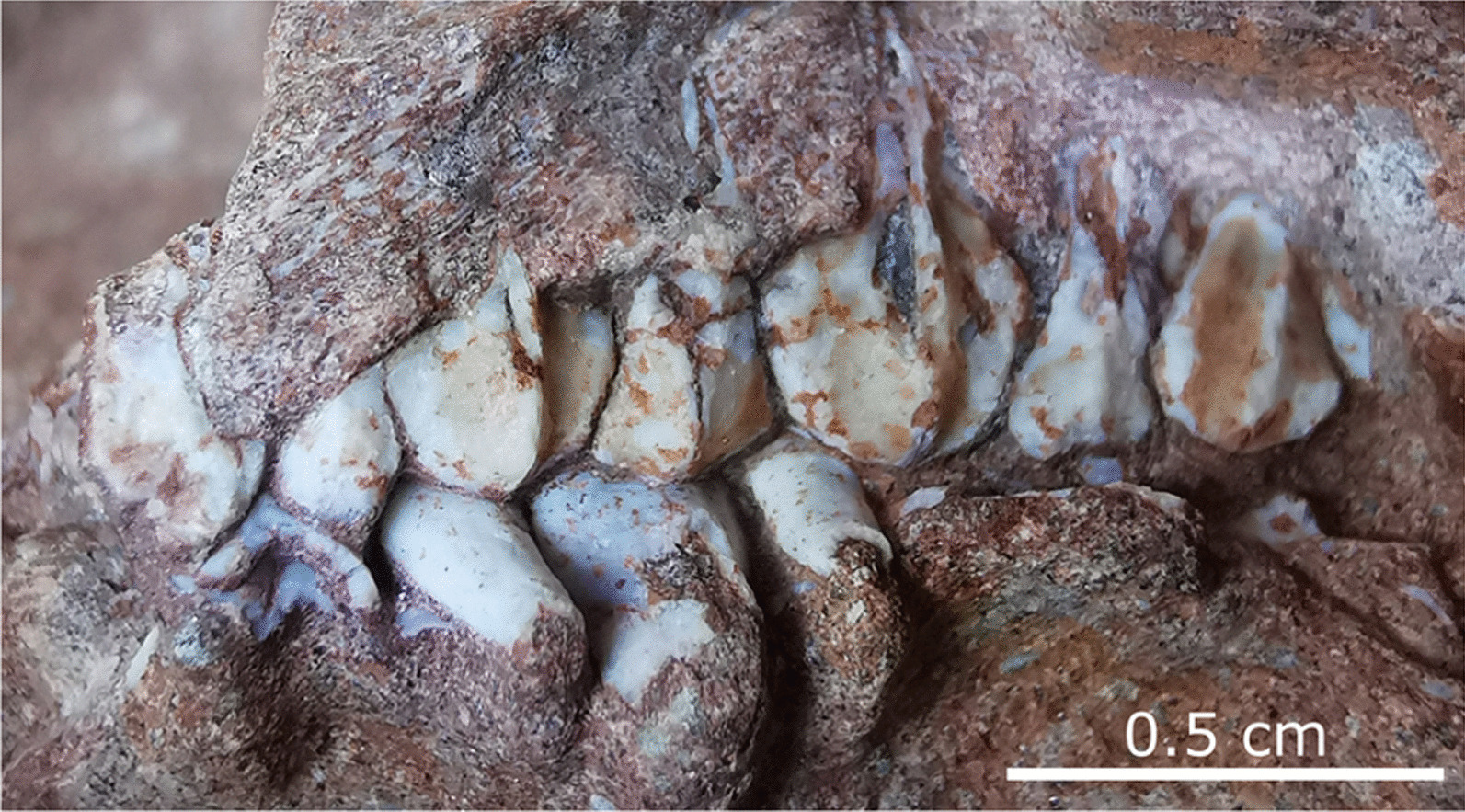
Occluded teeth of YLSNHM 01328. Maxillary teeth (top row) exhibit a strong primary (median) ridge and no subsidiary ridges or marginal denticles

This partial, articulated skeleton consists of the posterior cranium (missing most of the snout), complete cervical series, and the anterior-most dorsal vertebrae and associated ribs (Fig. 3). The bridge of the rostrum has buckled; its original profile is obscured. The elongate, paired nasals are unfused and broken anteriorly where they reach their greatest transverse breadth. In lateral profile, the nasal is subtly bowed dorsally above the naris. The squamosal has disarticulated from the postorbital. The postorbital process of the squamosal is tall, blunt, and constricts where it meets the main body of the element. The pre- and postcotylar processes are subequal in length. The squamosal compares most favourably with those of the hadrosauroids *Tanius sinensis* [35], *Levnesovia transoxiana* [36], and *Nanningosaurus dashiensis* [37]. By contrast, the postorbital process of the squamosal is much longer and slenderer in most other hadrosauroids, including *Gobihadros mongoliensis* [38], *Prosaurolophus maximus* [39], and *Corythosaurus casuarius* [40] (Fig. 4). The anterior third of the left maxilla is missing, whereas the preserved portion is 14 mm long. Whether a palatal process of the maxilla (sensu [41])—whose absence is diagnostic of Lambeosaurinae—originally existed cannot be determined. The jugal facet of the maxilla faces laterally, and its long axis is horizontal as in the perinates of *Maiasaura peeblesorum*, whereas the same facet is distinctly angled in those of *Hypacrosaurus stebingeri* [2]. The partial maxilla has eight teeth in situ (there is a total 12 maxillary teeth in embryonic *H. stebingeri*; [2]). Each has a straight primary (median) ridge, offset slightly distally, and lacks both subsidiary ridges and marginal denticles (Fig. 5), as in the perinates of *M. peeblesorum* [42]. The occlusal surfaces of the teeth are not visible, hindering determination of whether they bear wear facets as they do in the embryos of *H. stebingeri* [2]. The quadrate (17 mm tall) is robust with a broadly rounded head that articulates dorsally with the squamosal in a hinge joint. The pterygoid flange is broad, and the quadratojugal notch, largely obscured by the quadratojugal, occurs in the lower half of the quadrate body. The quadrate is gently bowed anteriorly along its length; the dorsal and ventral halves form an angle of approximately 154°. The ossified braincase elements are unfused and have been displaced; the basisphenoid now rests against the skull roof and the orbitosphenoid has shifted posteriorly. The prootic is visible beneath the basisphenoid, where the single opening for c.n. V (trigeminal nerve) can be seen. The otoccipital (opisthotic + exoccipital) occurs further posteriorly. On the occiput, the well-developed paroccipital process is pendant and projects posterolaterally beneath the squamosal.

The cervical series is nearly complete and preserved in a gentle sigmoid along its length, being dorsiflexed anteriorly and ventroflexed posteriorly. The neurocentral sutures are unfused. The neural spines are low, whereas the postzygapophyses are strongly developed and hooked, both of which are common to all hadrosauroids. The postzygapophyses do not extend above the level of the neural spines as they do in adult hadrosaurids (e.g., [43, 44]). Many of the transverse processes have buckled ventrally, obscuring their corresponding centra. The few remaining cervical ribs are L-shaped, with elongate posterior processes. The transition between the cervical and anterior dorsal series is nearly indistinguishable. We tentatively identify the first dorsal as that bearing the first preserved elongate rib, which itself is broken along its length. As such, we identify 12 cervical vertebrae, although the count may have been higher by one or two vertebrae (depending on whether the first preserved long rib reached the sternum or not). For comparison, the cervical series contains 11 vertebrae in most known non-hadrosaurid hadrosauroids [38, 45, 46], and varies between 12 and 18 vertebrae in hadrosaurids [6, 47]. The neural spines of YLSNHM 01328 increase in size and posterior inclination by the 15th presacral vertebra, but the dorsal series is not preserved beyond the 17th presacral vertebra.

#### YLSNHM 01373

**Fig. 6:**
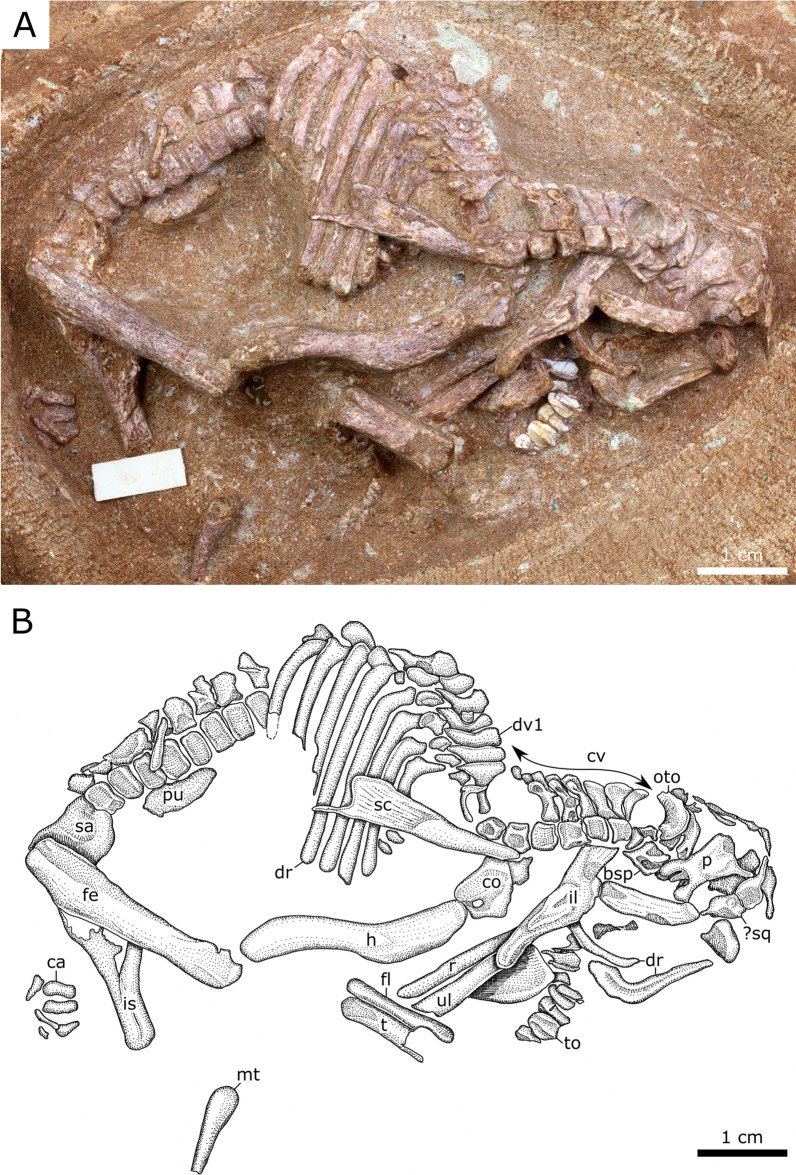
Hadrosauroid partial embryonic skeleton (YLSNHM 01373). **A** Photograph, **B** interpretive drawing. See text for list of abbreviations

This articulated skeleton is lacking parts of the skull, distal limb elements, and tail (Fig. 6). The parietal, which is poorly visible in YLSNHM 01328, is elongate (2.7 times longer than wide), as in non-lambeosaurine hadrosauroids. A partial tooth row has been displaced from the jaws and now lies adjacent to the similarly displaced left ilium. The dissociation of the teeth from their host element makes it difficult to determine whether they originated within the maxilla or dentary. The morphology of the tooth crowns agrees with that of the maxillary teeth of YLSNHM 01328. The preserved cervical and dorsal vertebrae show unfused neural arches. We consider the first dorsal vertebra to be that bearing the first long rib that presumably connected with the sternum, in which case, we count 11 cervical vertebrae and 18 dorsal vertebrae. However, we can neither confirm the presence of the atlas/axis in the preserved series nor rule out the possibility that the posteriormost free dorsal vertebra would eventually become incorporated in the synsacrum as a dorsosacral [6]; the true cervical count could be higher. The few preserved caudal vertebrae show that the neural spines were low. The coracoid foramen is not enclosed but opens posteriorly to separate the contact surfaces for the humerus and scapula, as in *Hypacrosaurus stebingeri* perinates [2]. The scapular blade is slender and the caudal end is irregular and poorly ossified. The length of the deltopectoral crest of the humerus is moderate compared to the length of the humerus (ratio = 0.53), which contrasts with the condition of most hadrosaurids (ratio > 0.55) [6, 7]. The distal condyles of the humerus are poorly defined. The left ilium has drifted anteriorly to be preserved alongside the skull. It is low in lateral profile, with a preacetabular blade that is only weakly arched dorsally. The pubic and ischiadic penduncles are poorly defined. The femur is 26 mm long, with a well-developed greater trochanter and poorly defined distal condyles. The fourth trochanter is not visible, on account of the outward rotation of the femur. The preserved tibia and fibula are missing their extremities.

## Discussion and conclusions

The embryos described here can be confidently assigned to Hadrosauroidea based on the following derived features (from [7]): (1) large jugal contact of maxilla faces strongly laterally; (2) deltopectoral crest of humerus wide relative to minimum width of humeral shaft (ratio = 1.71). Dentary teeth having a centrally located primary ridge and lacking subsidiary ridges are also considered derived for hadrosauroids [7], and while these features are present on the maxillary teeth of the embryos described here, we are unable to confirm their occurrence specifically on the dentary teeth (the dentary tooth crowns are not visible in YLSNHM 01328, and the loose teeth of YLSNHM 01373 are not definitively from the dentary).

Derived features that would allow the embryos to be assigned to Hadrosauridae (see synapomorphies listed in [7]) are lacking. For example, in hadrosaurid adults and embryos, the maxilla often features a well-defined ectopterygoid ridge [2, 36], which is missing in YLSNHM 01328. Hadrosaurids of all stages of development also possess a deltopectoral crest of the humerus that is both proximodistally elongate and exhibits a pointed distal corner [2, 5, 42, 48]; this is not the case in YLSNHM 01373. The parietal and nasals are also long compared to the condition seen in lambeosaurines (although parietal length is negatively allometric over the course of lambeosaurine ontogeny; [7]). Many definitive hadrosaurid characters are associated with the ilium. However, these characters typically relate to the pubic and ischial peduncles that bound the acetabulum, which is poorly preserved in YLSNHM 01373, and so these characters cannot be properly assessed. The relatively high cervical count (12 +) that we report in the embryos is consistent with that of most hadrosaurids [6], but cervical count does not unambiguously diagnose Hadrosauridae [7].

The squamosal of YLSNHM 01328 is distinctive in having a relatively tall, blunt postorbital process, which is also seen in the hadrosauroids *Levnesovia transoxiana*, *Nanningosaurus dashiensis*, and *Tanius sinensis* (Fig. 4). Further comparison with these taxa is complicated by the fact that none are very complete and that all are known from osteologically mature individuals. The holotype of *Levnesovia transoxiana* is represented by a partial skull and scattered postcranial elements [36]. Most notably, the teeth differ from those of the embryos described here in having denticulate crown margins and weak secondary ridges (the latter on the dentary teeth only). The holotype of *Nanningosaurus dashiensis* consists of scant skull and postcranial remains [37]. The dorsal process of the maxilla differs from that of YLSNHM 01328 in being sharply peaked. The maxillary teeth primarily differ from those reported here in being more numerous and narrower mesiodistally, both characters known to vary with age in the hadrosaurid *Hypacrosaurus stebingeri* [2]. The dentary teeth of *N. dashiensis* bear secondary ridges and marginal denticles, but these features cannot be confirmed in YLSNHM 01328 because the dentary tooth crowns are obscured by the overlying maxillary teeth. The isolated tooth row preserved with YLSNHM 01373 cannot be confidently ascribed to the dentary to facilitate comparison with *N. dashiensis*. The holotype of *Tanius sinensis* consists of a posterior cranium, varied appendicular bones, a series of ten cervical vertebrae (complete count unknown), and a few other axial elements [35]. No extant diagnosis exists for the cranium, and the postcranium is relatively conservative. The dorsal neural spines are purportedly tall for a non-hadrosaurid hadrosauroid [49], but neural spine height is known to increase with age in *H. stebingeri* [2]. The poor temporal resolution of the host Hekou Formation makes it difficult to determine whether the described embryos were penecontemporaneous with any of the other Late Cretaceous hadrosauroid taxa just mentioned (and whose temporal ranges are sometimes likewise poorly constrained). If we accept that the Hekou Formation is of Maastrichtian age (see ‘Geological provenance’ above), then the hadrosauroid embryos may only be penecontemporaneous with *T. sinensis*, which itself is from somewhere within the upper Campanian to lower Maastrichtian [49].The occurrence of hadrosauroids in the Hekou Formation is not unprecedented. Their presence was initially signaled following the description of large ornithopod tracks in Upper Cretaceous red beds elsewhere in southern China [50, 51]. More recently, Xing et al. [25] described a partial axial skeleton of a hadrosaurid from the Hekou Formation, identified on the basis of the long and robust postzygapophyses of the cervical vertebrae. Similarly developed processes are not present in the embryos described here, supporting the existence of at least two hadrosauroid taxa within the Hekou Formation—a hadrosaurid and a non-hadrosaurid hadrosauroid.

In describing hadrosaurine and lambeosaurine eggs and embryos from Montana, Horner [4] noted that those of hadrosaurines tend to be much smaller than those of lambeosaurines. Hadrosaurine embryos (*Maiasaura peeblesorum*) have femora that vary in length between 35 and 40 mm, and their subspherical eggs were calculated to have a volume of approximately 900 mL. Lambeosaurine embryos (*Hypacrosaurus stebingeri* and an indeterminate form), by contrast, have femora that vary between 60 and 80 mm long, and are derived from eggs approaching 4000 mL [2, 4]. Horner [4] hypothesized that these differences were typical of their respective subfamilies, and further suggested that the smaller hadrosaurine hatchlings were altricial, based on their poorly ossified epiphyses [3, 52].

**Fig. 7:**
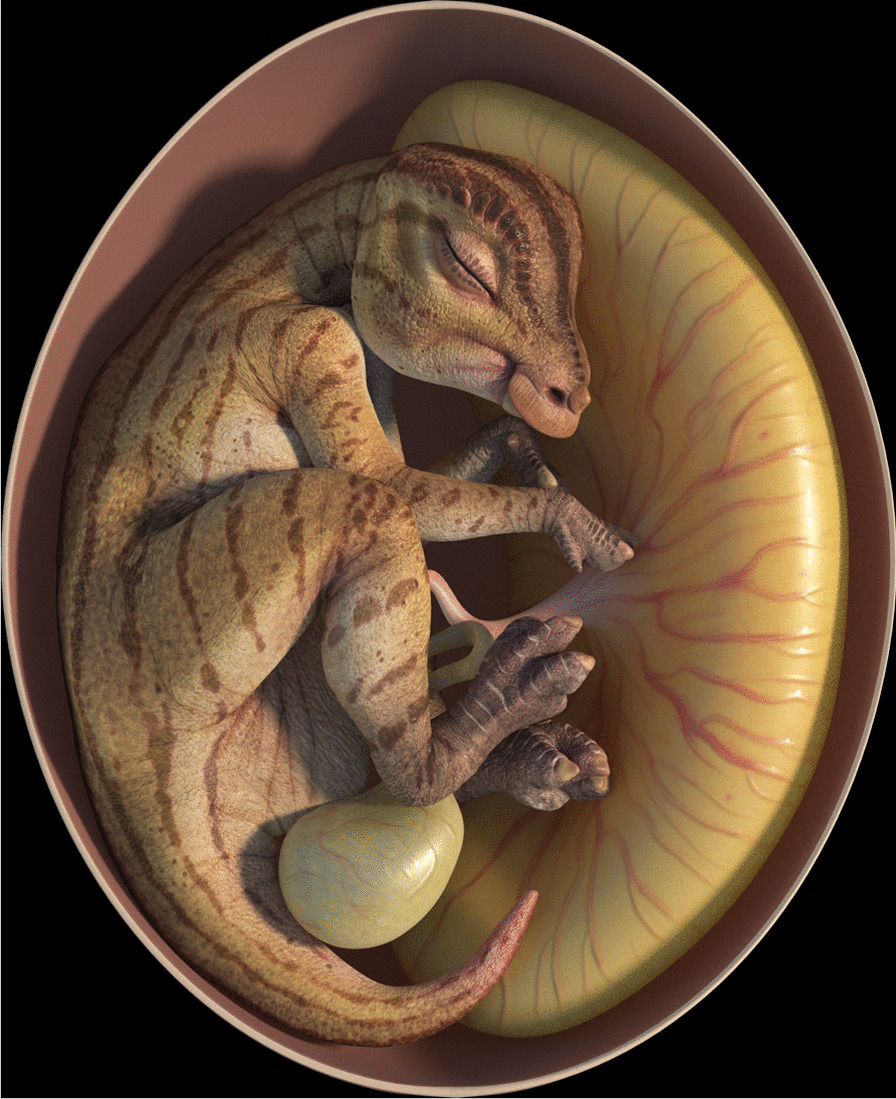
Hadrosauroid embryo, based on YLSNHM 01373

The hadrosauroid eggs and embryos reported here facilitate character polarization of egg and hatchling size among hadrosaurids. The 26 mm femur of YLSNHM 01373 is closer to *M. peeblesorum* perinates in size, and the corresponding egg (660 mL) similarly is more like those of *M. peeblesorum* than to those of any known lambeosaurine [4]. Importantly, the YLSNHM 01373 embryo is not fully developed, evidenced by the fact that the skeleton does not entirely fill the egg [cf. 54]. This likely explains the missing ends of the tibia-fibula, which ossify from the diaphyses outward, and the absence of many of the manual and pedal elements in an otherwise mostly undisturbed skeleton; they may simply not have ossified by the time of death [54]. It is probable, therefore, that these embryos were yet several embryonic stages away from hatching (Fig. 7). Consequently, we are unable to determine whether the corresponding neonates were altricial, given the incomplete development of the embryo. However, in the closely related *Telmatosaurus transsylvanicus*, the hatchlings are similarly small and their limb bone epiphyses are poorly formed [55], as in *M. peeblesorum*. These observations strongly imply that the lambeosaurine condition of having larger eggs and precocial hatchlings is an evolutionarily derived trait.

Dinosaur eggs and embryos commonly occur in semi-arid, upland palaeoenvironments [1]. Palaeontologists once maintained that these were the preferred nesting grounds of dinosaurs [32, 56], but rare perinatal bones have also been found in lowland deposits (e.g., [48, 57], and it is now widely held that the wet, acidic conditions of such palaeoenvironments exerted a bias against the preservation of eggs and their contents [57]. The Hekou Formation of China, with its diversity of fossil dinosaur eggs combined with sedimentological indicators of moderately dry, well-drained conditions (e.g., caliche, mudcracks, carbonate nodules), is entirely consistent with this framework of understanding. These strata promise to reveal many more clues about early ontogenetic development in dinosaurs.

## Methods

### Eggshell histology

A piece of eggshell was removed from YLSNHM 01373 with an Engraving Pen AT-310. The shell was embedded in Araldite 2020, cut with a STX-202A diamond wire cutting machine, and then polished with P400 to P4000 abrasive paper to approximately 30 μm thick for microscopic observation under normal and cross-polarized light with a Zeiss Primotech microscope.

## Data Availability

All data generated or analysed during this study are included in this published article.

## Abbreviations

atic: Atlas intercentrum
ax: Axis
bsp: Basisphenoid
ca: Caudal vertebrae
CL: Continuous layer
cnV: Cranial nerve V exit
co: Coracoid
cv: Cervical vertebrae
?d1: First dorsal vertebra (possibly)
dto: Dentary tooth
dr: Dorsal rib
dv: Dorsal vertebrae
f: Frontal
fe: Femur
fl: Fibula
h: Humerus
il: Ilium
is: Ischium
j: Jugal
mt: Metatarsal
ML: Mammillary layer
mx: Maxilla
mxto: Maxillary tooth
n: Nasal
oc: Occipital condyle
osp: Orbitosphenoid
oto: Otoccipital
p: Parietal
pl: Palatine
po: Postorbital
poc: Postorbital contact
pocp: Postcotyloid process
prcp: Precotyloid process
prf: Prefrontal
pro: Prootic
pt: Pterygoid
pu: Pubis
q: Quadrate
qj: Quadratojugal
r: Radius
sa: Sacrum
sc: Scapula
sp: Swollen process of squamosal
sq: Squamosal
t: Tibia
to: Tooth
ul: Ulna
YLSNHM: Yingliang Stone Natural History Museum
